# Quercetin Reduces Lipid Accumulation in a Cell Model of NAFLD by Inhibiting De Novo Fatty Acid Synthesis through the Acetyl-CoA Carboxylase 1/AMPK/PP2A Axis

**DOI:** 10.3390/ijms23031044

**Published:** 2022-01-18

**Authors:** Antonio Gnoni, Benedetta Di Chiara Stanca, Laura Giannotti, Gabriele Vincenzo Gnoni, Luisa Siculella, Fabrizio Damiano

**Affiliations:** 1 Department of Basic Medical Sciences, Neurosciences and Sense Organs, University of Bari “Aldo Moro”, 70121 Bari, Italy; antonio.gnoni@uniba.it; 2 Laboratory of Molecular Biology, Department of Biological and Environmental Sciences and Technologies, University of Salento, 73100 Lecce, Italy; benedetta.dichiara@unisalento.it (B.D.C.S.); laura.giannotti@unisalento.it (L.G.); luisa.siculella@unisalento.it (L.S.); 3 Laboratory of Biological Chemistry, Department of Biological and Environmental Sciences and Technologies, University of Salento, 73100 Lecce, Italy; gabriele.gnoni@unisalento.it

**Keywords:** acetyl-CoA carboxylase 1, AMP-activated protein kinase, de novo lipogenesis, endoplasmic reticulum stress, quercetin, citrate carrier, non-alcoholic fatty liver disease, protein phosphatase 2A, sterol regulatory element-binding protein 1

## Abstract

Dysregulation of de novo lipogenesis (DNL) has recently gained strong attention as being one of the critical factors that contribute to the assessment of non-alcoholic fatty liver disease (NAFLD). NAFLD is often diagnosed in patients with dyslipidemias and type 2 diabetes; thus, an interesting correlation can be deduced between high hematic free fatty acids and glucose excess in the DNL dysregulation. In the present study, we report that, in a cellular model of NAFLD, the coexistence of elevated glucose and FFA conditions caused the highest cellular lipid accumulation. Deepening the molecular mechanisms of the DNL dysregulation—RT-qPCR and immunoblot analysis demonstrated increased expression of mitochondrial citrate carrier (CiC), cytosolic acetyl-CoA carboxylase 1 (ACACA), and diacylglycerol acyltransferase 2 (DGAT2) involved in fatty acids and triglycerides synthesis, respectively. XBP-1, an endoplasmic reticulum stress marker, and SREBP-1 were the transcription factors connected to the DNL activation. Quercetin (Que), a flavonoid with strong antioxidant properties, and noticeably reduced the lipid accumulation and the expression of SREBP-1 and XBP-1, as well as of their lipogenic gene targets in steatotic cells. The anti-lipogenic action of Que mainly occurs through a strong phosphorylation of ACACA, which catalyzes the committing step in the DNL pathway. The high level of ACACA phosphorylation in Que-treated cells was explained by the intervention of AMPK together with the reduction of enzymatic activity of PP2A phosphatase. Overall, our findings highlight a direct anti-lipogenic effect of Que exerted through inhibition of the DNL pathway by acting on ACACA/AMPK/PP2A axis; thus, suggesting this flavonoid as a promising molecule for the NAFLD treatment.

## 1. Introduction

Non-alcoholic fatty liver disease (NAFLD) represents a major global public health challenge and it is the most common chronic liver disease worldwide. It ranges from simple steatosis without specific inflammatory behaviors to more severe nonalcoholic steatohepatitis (NASH) and cirrhosis, with a seriously increased risk of developing hepatocellular carcinoma [1]. In NAFLD, triglycerides (TG) are accumulated as lipid droplets in the cytoplasm. This accumulation is derived from several factors, including dietary fat ingestion, deregulated free fatty acids (FFAs) release by adipose tissue through lipolysis, and inadequate fatty acid oxidation [2,3]. Recently, it was shown that de novo lipogenesis (DNL), i.e., the de novo synthesis of fatty acids from carbohydrate sources (mainly glucose), accounts for at least 35% of lipids present in steatotic cells [4,5]. This aspect undoubtedly constitutes an evident paradoxical condition according to which new molecules of fatty acids are synthesized, while an abundance of lipids are available in steatotic cells.

The pathogenesis of NAFLD/NASH is quite complex, although a decisive role is played by insulin resistance (IR), which is frequently observed in the liver in obese patients with dyslipidemia and type 2 diabetes mellitus (T2DM) [6]. In IR conditions, hepatic DNL is stimulated through the activation of sterol regulatory element-binding protein-1 (SREBP-1), the main transcription factor implied in lipogenic gene upregulation in steatotic hepatocytes [4,5]. Under IR, insulin does not inhibit the synthesis of hepatic glucose, an anomalous condition that contributes to T2DM development. Together with the activation of SREBP-1, a high concentration of hematic glucose in T2DM triggers DNL and fatty acids conversion in TG; thus, causing lipid droplets accumulation in hepatocytes. Consequently, in obese and diabetic patients, IR sets up a vicious cycle, promoting the development of both NAFLD and T2DM [7].

Besides IR, the condition of endoplasmic reticulum (ER) stress and the activation of the unfolded protein response (UPR) pathway have been found as markers of NAFLD [8,9,10,11,12]. Several pieces of evidence suggest a strict correlation between the increased lipogenesis in NAFLD and the induction of the transcriptional factor XBP-1, which represents one among the ER stress/UPR effectors [8,9,10,11,12].

In NAFLD, the increased lipid burden is also responsible for increased mitochondria activity (β-oxidation of free fatty acid, adenosine triphosphate (ATP) production, reactive oxygen species (ROS) generation), and mass [13]. Over time, mitochondria may become exhausted, leading to uncoupling, with an increase in oxidative stress due to increased ROS formation and impaired hepatic insulin resistance, thus favoring NASH development [13]. Mitochondrial ROS production plays a leading role in propagating hepatocyte damage through lipid peroxidation products and tumor necrosis factor-α production, both of which booster of mitochondrial injury, permeability, and uncoupling [13].

Lifestyle-based interventions and greater adherence to the Mediterranean diet (MD) are the main recommendations for tackling IR, obesity, and NAFLD [14,15,16].

Since NAFLD is a disease associated with unbalanced diets, the first intervention to tackle this pathology is the adoption of a correct lifestyle and the intake of healthy diets, for example, the Mediterranean diet rich in unsaturated fatty acids and polyphenols.

Quercetin (3,3′,4′,5,7-pentahydroxyflavone, Que) is a flavanol abundant in several foods in the MD, such as vegetables and fruits, as well as in red wine [17]. Its structure consists of two benzene rings and an oxygen-containing heterocycle, with several hydroxyl groups. This compound has a potent antioxidant effect with free radicals scavenging property, which is related to its molecular structure [17]. Several biological effects, including the improvement of IR and the reduction of obesity, have been ascribed to the intake of quercetin [17,18]. The beneficial properties of Que have been correlated to its anti-inflammatory activity, which plays a role in protecting against diabetes, obesity, cardiovascular and neurodegenerative diseases [13,19].

In high-fat diet (HFD)-fed rats, Que suppresses adipogenesis and reduces lipogenesis [20], whereas it decreases both DNL and TG synthesis in primary rat hepatocytes [21]. Que also elicits beneficial effects versus cancer development by inhibiting cholesterol and fatty acid synthesis [18]. Only a few (and not thorough) studies have focused on the Que effect on lipogenesis in NAFLD [22,23].

The aim of the present work was to thoroughly analyze the effect of Que in modulating the lipid synthesis pathway by using a widely used experimental steatotic model obtained by incubating HepG2 cells with 0.75 mM FFA mixture containing 2:1 oleate:palmitate [4,24]. Since hyperglycemia is often found in obese patients with NAFLD, the role of glucose as a distinct NAFLD stressor has been here evaluated, using two different glucose concentrations in the culture medium, 5.5 mM (low glucose, LG) and 25 mM (high glucose, HG). We observed that incubation of HepG2 cells with FFAs induced accumulation of lipid droplets, which was remarkably pronounced when cells were cultured in HG medium. This accumulation was observed even in the absence of exogenous FFAs, suggesting a causative role of glucose alone in triggering NAFLD.

Deepening the molecular mechanism at the basis of the intracellular lipid accumulation, we found that the simultaneous co-existence of elevated concentration of glucose and FFAs is the condition in which the highest activation of the XBP-1 and SREBP-1 lipogenic transcriptional factors was observed. Moreover, the highest expression of citrate carrier (CiC), a mitochondrial protein representing a link between carbohydrate and lipid metabolism, of cytosolic acetyl-CoA carboxylase 1 (ACACA), as well as of diacylglycerol o-acyltransferase 2 (DGAT2), which are deputed in FFAs and TG synthesis, respectively, was observed. These effects were efficaciously counteracted by the addition of Que in the medium. We also provide evidence that the anti-lipogenic mechanism of Que was explained primarily by the strong phosphorylation of ACACA, which catalyzes the committing step in the DNL pathway. The strong phosphorylation of ACACA might be only partially explained by the Que-mediated intervention of AMPK. Indeed, a remarkable reduction of the enzymatic activity of PP2A phosphatase was observed in lipid droplet-overloaded cells following incubation with Que. Overall, the findings presented here depone for a direct anti-lipogenic effect of Que on the DNL pathway through molecular mechanisms of Que exerted on ER stress and ACACA/AMPK/PP2A axis.

## 2. Results

### 2.1. Que Treatment Attenuated Lipid Accumulation in FFA-Treated HepG2 Cells

**Figure 1:**
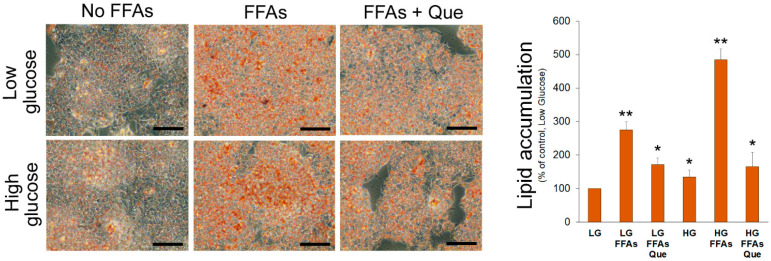
Effects of Que on lipid accumulation in HepG2 cells. Representative pictures of lipid droplets accumulation in cells incubated in the low glucose (LG) or high glucose (HG) medium, with or without the oleate:palmitate 2:1 mixture and 5 µM Que. After Oil Red O staining, the incorporated stain was solubilized and quantified by a spectrophotometer at 510 nm. Values were reported in histograms as the percentage with respect to the control, represented by HepG2 incubated in LG without FFAs and Que. Results are representative of three different experiments. * *p* < 0.05 versus control (LG); ** *p* < 0.01 versus control (LG). The bars in micrographs correspond to 100 µm.

A 24 h incubation with FFAs caused accumulation of TG in cells cultured in LG medium (Figure 1). Consistent with the uptake and use of glucose as a carbon source for lipogenesis, a more evident lipid accumulation was observed in HepG2 cells cultured in HG medium even in the absence of exogenous FFAs (Figure 1). However, the incubation of cells with the combination of high glucose and FFAs caused the most remarkable accumulation of lipid droplets in the cytosol, suggesting that HG triggers DNL and potentiates the intracellular accumulation of lipid droplets. Incubation with 5 µM Que reduced lipid droplet accumulation in steatotic HepG2 cells incubated in LG and especially in HG media.

### 2.2. Que Treatment Attenuated Endoplasmic Reticulum Stress Triggered by Lipid Accumulation

**Figure 2:**
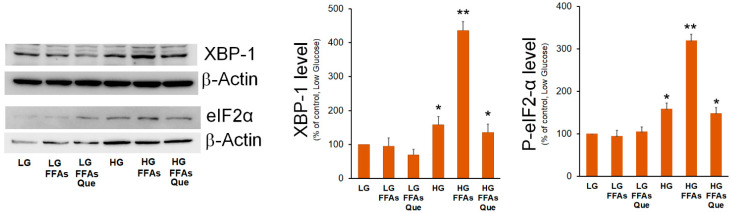
Effects of FFAs and Que treatment on the expression of ER stress marker. HepG2 cells were incubated in low glucose (LG) or in high glucose (HG) medium, in the presence or in the absence of 0.75 mM FFAs and 5µM Que for 24 h. Total proteins were extracted from the cells and separated by SDS/PAGE. After incubation with antibodies against XBP-1 or P-eIF2α, the content of XBP-1 and P-eIF2α was quantified by densitometric analysis and expressed as a percentage with respect to control cells (LG). Values are means ± S.D. Results are representative of three different experiments. * *p* < 0.05 versus control (LG); ** *p* < 0.01 versus control (LG).

Two branches of UPR, i.e., the IRE1α/XBP-1 and PERK/eIF2α, were previously demonstrated to be involved in increased lipogenic genes expression during ER stress and in NAFLD [25]. Previously, we demonstrated that the XBP-1 transcription factor stimulates the expression of the mitochondrial citrate carrier [26], which provides the precursors for DNL. Here, we evaluated the effects of the treatment with FFAs and Que on the expression of XBP-1. HepG2 cells cultured in the HG medium showed XBP-1 levels higher than those observed in LG-treated cells (Figure 2). However, when cells were cultured in HG-FFAs the increase of XBP-1 levels was much higher with respect to cells incubated in HG alone. By contrast, no significant change was observed between LG and LG-FFAs treated cells. When compared with HG-FFAs sample, a dramatic reduction of XBP-1 expression was observed upon the treatment with Que (Figure 2).

EIF2α is the alpha subunit of the translation initiation factor eIF2 involved in the canonical cap-dependent initiation of translation. Phosphorylation of eIF2α causes the downregulation of the global cap-dependent translation. The results of Western blotting experiments showed that eIF2α phosphorylated form was significantly increased in HG medium, even though its highest level was detected in HG-FFAs cells. Treatment with Que reduced eIF2α phosphorylation to the level seen in HG cells (Figure 2).

### 2.3. Que Treatment Reduced Lipogenic Genes Stimulation in FFAs-Treated HepG2 Cells

**Figure 3:**
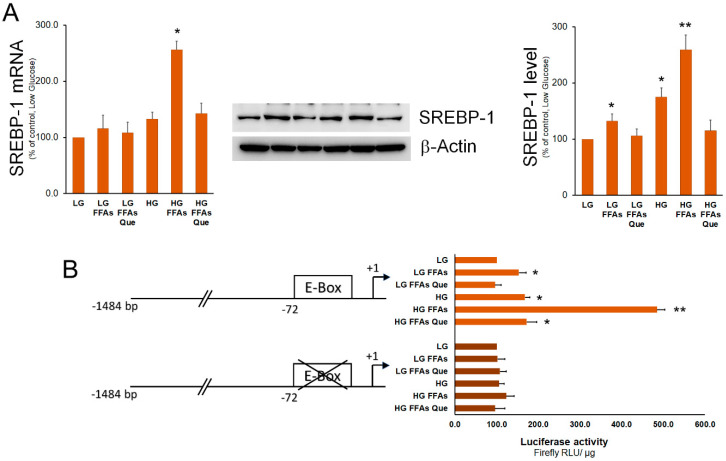
Effects of FFAs and Que treatment on the expression and trans-activation activity of SREBP-1. (**A**) HepG2 cells were incubated in low glucose (LG) or in high glucose (HG) medium, in the presence or in the absence of 0.75 mM FFAs and 5µM Que for 24 h. Total proteins were extracted from the cells and separated by SDS/PAGE. After incubation with antibodies against SREBP-1, the content of SREBP-1 was quantified by densitometric analysis and expressed as a percentage of control cells (LG). Values are means ± S.D. Results are representative of three different experiments. * *p* < 0.05 versus control (LG); ** *p* < 0.01 versus control (LG). (**B**) Cells were transiently co-transfected with the CiC promoter-luciferase constructs together with Renilla luciferase reference plasmid pGL4.73. After 24 h, HepG2 cells were incubated in low glucose (LG) or in high glucose (HG), in the presence or in the absence of 0.75 mM FFAs and 5µM Que for 24 h, and firefly luciferase activity was measured and normalized to Renilla luciferase activity and to protein concentration. CiC promoter was expressed as a percentage of the control cells (LG). Values are means ± S.D. Results are representative of five different experiments. * *p* < 0.05 versus control (LG); ** *p* < 0.01 versus control (LG).

SREBP-1 represents the master transcription factor regulating the expression of genes involved in fatty acid and TG synthesis [27]. Here, we show that, when compared to control cells (LG medium), the expression of SREBP-1 was higher in HepG2 cells cultured in the HG medium. Treatment of HepG2 cells with FFAs determined a strong increase of SREBP-1 expression in cells incubated in the HG medium and, to a lesser extent, in the LG medium (Figure 3). In both LG and HG media, Que treatment caused a rescue of SREBP-1 expression at the level of the respective control cells. To demonstrate whether variations in SREBP-1 levels corresponded to an alteration in its transactivation activity, a luciferase assay was performed. To this purpose, the pCiC1484-Luc construct with the promoter region of the Cic gene containing a characterized SREBP-1 binding site (E-box) was employed [28]. As a negative control, the p72Em construct containing the same promoter region and the mutated SREBP-1 binding site was used. Results obtained indicated that the transactivation activity of SREBP-1 was increased in steatotic compared to control cells grown in LG. This increase was much more evident in steatotic cells grown in HG than in those grown in LG. When compared to steatotic cells, the transactivation activity of SREBP-1 was found to be reduced by Que treatment to the level observed in control cells.

**Figure 4:**
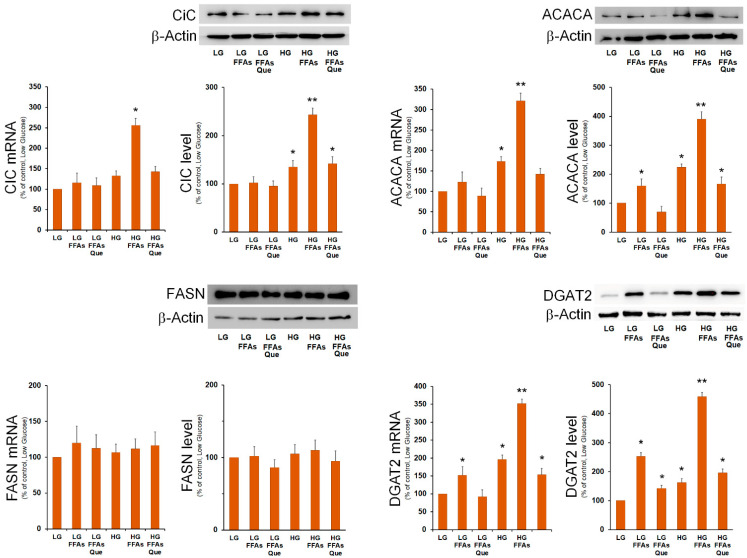
Effects of FFAs and Que treatment on the expression of genes involved in fatty acid and triglyceride synthesis. HepG2 cells were incubated in low glucose (LG) or in high glucose (HG) medium, in the presence or in the absence of 0.75 mM FFAs and 5 µM Que for 24 h. Total proteins were extracted from the cells and separated by SDS/PAGE. After incubation with antibodies against CiC, ACACA, FASN, and DGAT2, the content of each protein was quantified by densitometric analysis and expressed as percentage of control cells (LG). Values are means ± S.D. Results are representative of three different experiments. * *p* < 0.05 versus control (LG); ** *p* < 0.01 versus control (LG).

Then, we analyzed the expression of SREBP-1-responsive lipogenic genes involved in DNL and in TG synthesis. We found that the mRNA amount and the protein level for mitochondrial citrate carrier (CiC), acetyl-CoA carboxylase 1 (ACACA), and diacylglycerol O-acyltransferase 2 (DGAT2) were remarkably augmented in cells incubated in HG and FFAs (Figure 4). The expression of fatty acid synthase (FASN) did not seem to be influenced by lipid accumulation. The expression of SREBP-1-target genes Cic, Acaca, and Dgat2, the key enzyme involved in triglycerides synthesis [21], were strongly reduced by Que treatment, with respect to the respective steatotic cells in LG and HG media (Figure 4).

### 2.4. Que Treatment Strongly Inhibits Acetyl-CoA Carboxylase Activity

The findings obtained so far indicated that treatment with quercetin was effective in reducing the lipid accumulation, through a reduction of the expression of SREBP-1 transcription factor as well as of its lipogenic target genes. Among the different regulatory mechanisms of the activity of acetyl-CoA carboxylase 1 (ACACA), which catalyzes the first committed step in fatty acid biosynthesis, the phosphorylation on its serine 79 (Ser79) residue represents a crucial point of DNL regulation by hormones and nutrients.

**Figure 5:**
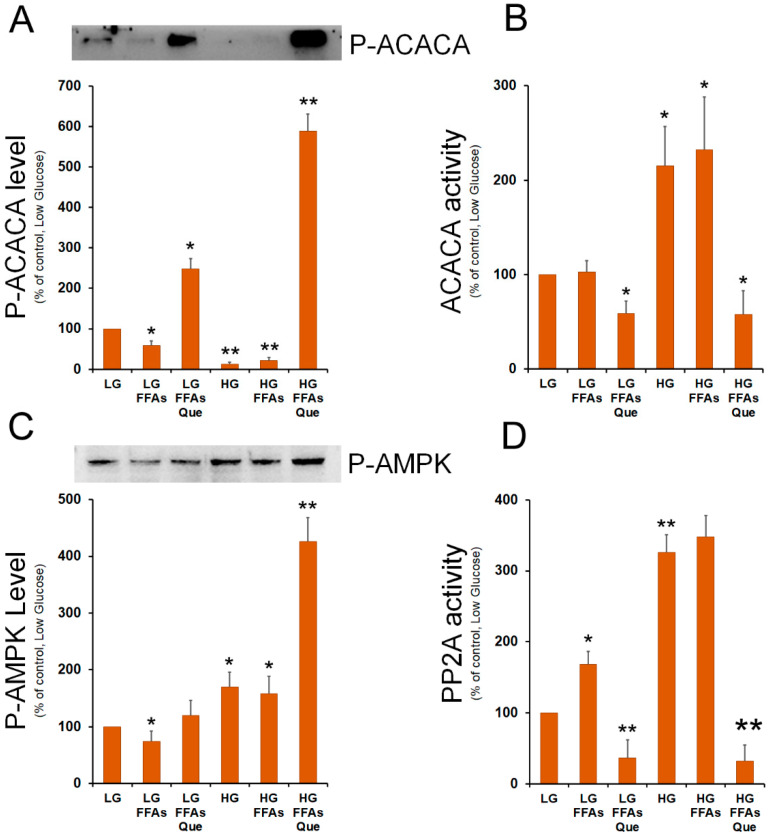
Role of FFAs and Que on the regulation of ACACA activity. (**A**) HepG2 cells were incubated in low glucose (LG) or in high glucose (HG) medium, in the presence or in the absence of 0.75 mM FFAs and 5 µM Que for 24 h. Total proteins were extracted from the cells and separated by SDS/PAGE. After incubation with antibodies against phosphorylated ACACA, the protein content was quantified by densitometric analysis, and results were expressed as a percentage of control cells (LG). Results are representative of three different experiments. * *p* < 0.05 versus control (LG); ** *p* < 0.01 versus control (LG). Values are means ± S.D. (**B**) Cells were treated as reported in A. ACACA activity was determined in cell lysates as in Material and Methods. The activity in each sample was expressed as a percentage of control cells (LG). Values are means ± S.D., *n* = 3. * *p* < 0.05 versus control (LG); ** *p* < 0.01 versus control (LG). (**C**) After the separation of cell lysates by SDS/PAGE, proteins were transferred onto nitrocellulose membrane and blotted against phosphorylated AMPK. The protein content was quantified by densitometric analysis and expressed as a percentage of control cells (LG). Results are representative of three different experiments. * *p* < 0.05 versus control (LG); ** *p* < 0.01 versus control (LG). (**D**) PP2A activity was determined in cell lysates as reported in Material and Methods. The activity in each sample was expressed as a percentage of control cells (LG). Values are means ± S.D., *n* = 3. * *p* < 0.05 versus control (LG); ** *p* < 0.01 versus control (LG).

Indeed, AMP-activated protein kinase (AMPK) phosphorylates ACACA on Ser79 causing the inhibition of the ACACA enzymatic activity. By contrast, ACACA enzymatic re-activation through its de-phosphorylation is catalyzed by protein phosphatase 2 (PP2A). Therefore, we investigated the effect of lipid accumulation and Que treatment on the level of p-ACACA (Ser79). Western blots showed that p-ACACA (Ser79) level was reduced in steatotic cells incubated in LG or HG medium, while it was strongly evident in Que-treated cells, mainly in cells grown in HG medium (Figure 5A). Enzymatic assay confirmed that, when compared to untreated cells, a strong inhibition of ACACA activity was observed in Que-treated cells (Figure 5B).

To give an explanation of the different content of P-ACACA in the various samples, we evaluated the level of phosphorylated AMPK, which is the ACACA specific kinase. When compared to the untreated cells, Que treatment caused an increase in the level of P-AMPK, which was more evident in HG-FFAs treated cells (Figure 5C). In these cells, we also evaluated the putative role of PP2A in raising the levels of P-ACACA. Western blotting experiments performed with the antibodies against the regulatory (PP2A-B) and the catalytic (PP2A-C) subunits of PP2A, showed that the expression of PP2A-B and PP2A-C remains unchanged among the samples (Supplementary Figure S1). Next, we investigated the PP2A specific phosphatase activity in the protein extracts from the different samples. We observed that FFA treatment caused an increase in PP2A activity in LG-growth protein extract. Incubation of cells in the HG medium caused a further increase of PP2A activity with respect to the LG sample (Figure 5D). The highest PP2A activity was detected in HG-FFA treated cells. Interestingly, treatment with Que caused a remarkable inhibition of PP2A activity compared to LG control cells (Figure 5D).

## 3. Discussion

NAFLD affects a large part of the population worldwide [29] and it is mainly associated with an unbalanced Western diet, rich in saturated fatty acids and poor in vegetables, fruits, and fish [30].

Since NAFLD is a disease associated with unbalanced diets, the first intervention to tackle this pathology is the adoption of a correct lifestyle and the intake of healthy diets, rich in fruits and vegetables, which are abundant in polyphenols, such as quercetin. Referring to the effects of Que on NAFLD, few and fragmentary data are available from the literature.

In this study, we investigated the effect of Que on steatotic cells and the molecular mechanisms by which Que determines the reduction of lipid accumulation in a cellular model of NAFLD. Several and different in vitro models, primary or stabilized hepatocytes incubated with FFAs, have been used in NAFLD studies [24]. Even though these models cannot replace the in vivo ones, they offer the possibility of studying specific molecular mechanisms under controlled experimental conditions, so the role of a single factor in determining the fatty liver can be evaluated [24]. The in vitro model applied in this study (HepG2 incubated with 0.75 mM oleate/palmitate in 2:1 ratio) aims to imitate benign chronic steatosis, without significant apoptotic effects which have been observed when palmitate alone is used [24,31]. Since in human NAFLD coexists with other metabolic disorders, in particular, type 2 diabetes [32], we also investigated the influence of glucose as a further and distinct parameter for evaluating the effect of FFA and Que on lipid accumulation.

The results here presented show that the HepG2 cells grown in LG exhibited accumulation of lipid droplets when treated with FFA alone, supporting the role of exogenous FFA in causing steatosis. The accumulation of lipid droplets is remarkably evident in the cells incubated in the culture medium without FFA, but containing a high concentration of glucose. This observation is well justified by the function of hepatocytes to convert excess glucose into fatty acids through glycolysis and DNL. When HG and FFAs were in the same culture medium, the accumulation of lipids was more evident, suggesting a clear synergistic effect of the single factors in causing NALFD. Importantly, Que was able to reduce the accumulation of lipid droplets induced by exogenous FFAs in both LG- and HG-treated cells.

To deepen the molecular mechanism of this lipid accumulation, we decided to investigate the expression of enzymes involved in the lipogenic pathway, CiC, ACACA, FASN, and DGAT2. CiC, also known as tricarboxylate carrier, is an inner mitochondrial membrane protein that transports acetyl-CoA, mainly deriving from glucose, from mitochondria to the cytosol, where it represents the starter molecule for DNL. Thus, CiC represents a link between carbohydrate and lipid metabolism [33]. Apart from FASN, whose expression remained unchanged, we found that the expression of CiC, ACACA, and DGAT2 was increased in cells incubated with HG, supporting the conversion of excess glucose into fatty acids and TG. The highest expression of lipogenic genes has been reached in cells grown in the HG medium following the addition of FFAs. Moreover, the expression of SREBP-1 and its gene transactivation activity were strongly triggered by glucose and FFAs, highlighting the involvement of this lipogenic transcription factor in the regulation of the pathway of DNL in the NAFLD model. These results clearly imply that the maximal activation of the lipogenic pathway in the NAFLD model was triggered in the condition of high glucose and FFAs content in the medium and that each stimulus alone (HG or FFAs) was not sufficient to stimulate lipogenesis of the same magnitude.

Previous reports have highlighted the ER stress condition in the etiology of NAFLD [34], whereby SREBP-1 expression is strongly promoted through an efficient CAP-independent protein synthesis initiation [4]. Two branches of UPR, i.e., IRE1/XBP-1 and PERK/eIF2α, are strictly correlated to the lipogenic pathway induction during ER stress [8,26,35]. We found that a remarkable increment of XBP-1 and of P-eIF2α, has been observed in the HG-FFAs sample, which fosters the up-regulation of CiC and SREBP-1, as previously reported [11,26]. We also observed that incubation with HG or FFAs alone was not sufficient to induce XBP-1. Note that the addition of 5 µM Que to the culture medium was effective in reducing the ER stress, and thus the expression of SREBP-1 and XBP-1 transcription factors.

ACACA catalyzes the regulatory step of fatty acid synthesis and it is allosterically stimulated by citrate. Since we found CiC highly expressed in HG-FFAs treated cells, it can be assumed that the levels of citrate also increased under the same experimental conditions, thus triggering ACACA activity. In addition to the citrate-mediated positive regulation, ACACA is negatively regulated by its phosphorylation at the Ser79 residue, a post-translational modification mediated by the AMP-dependent protein kinase (AMPK). We found that incubation with high glucose (HG and HG-FFAs) greatly reduced the P-Ser79-ACACA amount, suggesting a putative role of HG in downregulating the level of ACACA phosphorylation. However, incubation with Que was effective in increasing the phosphorylation level of ACACA, thus nullifying the ACACA stimulation by glucose. The strong increase in the P-Ser79-ACACA amount can be partially justified by an increase in the P-AMPK level observed in the Que-treated HG-FFAs sample compared to the HG-FFAs sample. Indeed, AMPK phosphorylation is also observable in the HG sample, like that in Que-treated HG-FFAs. This discrepancy could be justified by the presence of other unknown kinases acting on ACACA differently regulated by HG, FFAs, and Que, a hypothesis that requires a dedicated study.

The high level of P-ACACA observed in the Que-treated HG-FFAs sample can be also explained by an alteration in the activity of the PP2A, the phosphatase involved in the dephosphorylation of P-ACACA. Analysis by Western blotting did not show significant changes in the levels of the catalytic and regulatory subunits of PP2A among all the samples. Conversely, the in vitro dephosphorylation assays indicate a strong reduction in the PP2A-mediated dephosphorylation activity in the samples treated with Que compared to the HG and HG-FFAs samples. The differences found in the PP2A phosphatase activity suggested the involvement of other regulatory mechanisms, like post-translational modifications of PP2A subunits or enzymatic modulation by endogenous molecules. It has been reported that ceramide, which synthesis depends on the palmitate level, activates PP2A [36] and induces IR by altering the insulin signaling pathway [37]. The highest lipogenic gene expression, together with the enhanced ACACA activity observed in the HG and HG-FFAs samples, could therefore lead to the synthesis of palmitate, main product of DNL and a component of ceramide. This could justify the higher PP2A activity observed in these samples with respect to LG samples. Another interesting hypothesis is that Que determines a direct inhibition of PP2A by binding its catalytic site. A similar hypothesis has been suggested from the in-silico docking study to explain the inhibition of PP2A activity by the polyphenol hesperidin, which has a structure very similar to that of Que [38].

Overall, this work highlights the importance of the combination of two distinct factors, i.e., the high concentration of glucose and the presence of free fatty acids, in determining the increase in lipid droplets in NAFLD. This aspect would then be considered in investigating the high prevalence of hepatic steatosis among obese and type 2 diabetic patients. Moreover, the same experimental condition (HG-FFAs) is the most effective in determining the consistent activation of the expression of lipogenic genes, correlated with the activation of lipogenic transcription factors XBP-1 and SREBP-1. From this study, it also emerges that, besides its nuclear action in reducing the expression of factors XBP-1 and SREBP-1, Que acts by regulating the activity of the ACACA, the main regulatory enzyme of the lipogenic pathway, whereby the axis AMPK/PP2A plays an important role in the control of phosphorylated-ACACA level. Note that in our experimental model Que was added after the induction of steatosis, indicating that this molecule is effectively capable of reversing the stimulation of lipogenesis and the intracellular accumulation of TG. The results obtained in this study open a new perspective of further investigation in humans to confirm the potential pharmacological use of Que in the treatment of NALFD.

## 4. Materials and Methods

### 4.1. Cell Culture Conditions, Treatment with Fatty Acids and Quercetin, and Triglycerides Content Determination

HepG2 were maintained in DMEM (Dulbecco’s modified Eagle’s medium) containing 4500 mg/Lt glucose (High Glucose, HG) (D5796, Sigma-Aldrich, Milano, Italy), supplemented with 10% (*v*/*v*) heat-inactivated fetal bovine serum (FBS), penicillin G (100 units/mL) and streptomycin (100 μg/mL), and kept at 37 °C in a humidified atmosphere containing 5% CO_2_. For each experimental condition, six 100 mm dishes were seeded with 10^6^ cells each in HG medium. After 24 h the culture medium from three dishes was replaced with fresh HG medium, whereas cells in the other three dishes were incubated in DMEM with 1000 mg/Lt glucose, here referred as DMEM-low glucose (LG). The cells were incubated for further 24 h. On the third day, lipid accumulation in cells was induced by adding 0.75 mM of a mixture of oleate/palmitate 2:1 (FFAs) dissolved in fatty acid-free BSA [4]. The molar ratio of FFAs to albumin was 4:1. BSA without FFAs was used as control experiments. A solution of 10 mM Quercetin was prepared in DMSO. Cell treatment with 5 µM Que was performed for 24 h by adding the compound to the culture medium (LG-FFAs or HG-FFAs). Determination of triglycerides content was determined using commercial kits (Randox Laboratories, Rome, Italy)).

For intracellular lipid droplet staining, HepG2 cells were grown at an initial density of 3 × 10^5^ cells/well in a 6-well plate and treated as described above. Cells were then washed three times with iced PBS and fixed with 10% formalin for 1 h. After fixation, cells were washed by 60% isopropanol and stained with Oil Red O solution (working solution, 0.3 g Oil Red O powder in 60% isopropanol) for 15 min at room temperature. To remove unbound staining, cells were rinsed once with 60% isopropanol and 4–5 times with distilled water. To quantify Oil Red O content levels, dimethyl sulfoxide was added to each sample; after shaking at room temperature for 5 min, the density of samples was read at 510 nm on a spectrophotometer.

### 4.2. Isolation of RNA from Cultured Cells and Real-Time qPCR Analysis

Total RNA from HepG2 cells was isolated using the SV Total RNA Isolation System kit (Promega Italia, Milano, Italy), following the manufacturer’s instructions. The reverse transcriptase (RT) reaction (20 μL) was carried out using 5 μg of total RNA, 100 ng of random hexamers and 200 units of SuperScript™ III RNase H-Reverse transcriptase (Life Technologies, Milano, Italy). Quantitative gene expression analysis was carried out on CFX Connect™ Real-Time PCR Detection System (Bio-Rad Laboratories, Segrate, Italy), using 18S rRNA for normalization. The primers used for real-time PCR analysis were listed in Table 1.

### 4.3. Western Blot Analysis

To obtain whole protein cell extracts for Western blot analysis, cells were scraped in the following buffer: 20 mM Tris-HCl, pH 8, 420 mM NaCl, 2 mM EDTA, 2 mM Na_3_VO_4_, and 1% (*v*/*v*) Nonidet P-40, supplemented with a cocktail of protease inhibitors. Cells were then passed several times through a 20-gauge syringe and centrifuged at 16,000× *g* for 20 min at 4 °C. The concentration of proteins in cell extracts was determined by the Bio-Rad protein assay kit, using lyophilized BSA for the calibration curve. 15–30 µg of total cell proteins were dissolved in sodium dodecyl sulfate (SDS) sample buffer and separated on 10% (*w*/*v*) SDS gels. Separated proteins were transferred onto nitrocellulose membrane (Pall, East Hills, NY, USA) [39]. Equal protein loading was confirmed by Ponceau S staining. The blots were blocked with 5% (*w*/*v*) non-fat dried milk in buffered saline and incubated with specific primary antibodies against FASN (610963 BD Biosciences, Milano, Italy), ACACA, phosphorylated-Ser79 acetyl-CoA carboxylase 1, PP2A-B56, demethylated-PP2A-C, DGAT1, CiC, XBP-1, SREBP-1, β-actin (sc-137104, sc-271965, sc-6954, sc-374380, sc-13601, sc-271934, sc-86392, sc-7160, sc-13551, sc-47778, Santa Cruz Biotechnologies, Dallas, TX, USA). The immune complexes were detected using appropriate peroxidase-conjugated secondary antibodies and enhanced chemiluminescent detection reagent (Western Bright ECL HRP substrate Advansta, San Jose, CA, USA). Densitometric analysis was carried out on the Western blots using the NIH Image 1.62 software (National Institutes of Health, Bethesda, Rockville, MD, USA), normalizing to β-actin used as a control.

### 4.4. Determination of ACACA and PP2A Enzymatic Activity

Cells were lysed in co-IP lysis buffer (10 mM Tris-HCl pH 7.4, 50 mM NaCl, 1 mM EGTA, 1 mM EDTA, 1 mM PMSF, 10 µg/mL leupeptin, 10 µg/mL aprotinin, 10 µg/mL pepstatin A) at 4 °C. The lysates were centrifuged at 16,000× *g* for 15 min at 4 °C, and the supernatants were incubated with 2.5 μg of anti-PP2A antibody for 2 h, followed by incubation with protein G agarose for one hour at 4 °C. The immunoprecipitates were washed once with co-IP lysis buffer, once with 50 mM Tris buffer (50 mM Tris, pH 7.5; 0.1 mM CaCl_2_), resuspended in assay buffer (50 mM Tris, pH 7.5; 0.1 mM CaCl_2_; 2.5 mM NiCl_2_, and 1 mg/mL p-nitrophenyl phosphate), and incubated at 37 °C for 30 min. The reaction was stopped by the addition of 13% K_2_HPO_4_, and the absorbance was read at 405 nm.

### 4.5. Statistical Analysis

Values are expressed as mean ± SD for the number of experiments indicated in the legends to the figures. Differences between the groups were determined by unpaired Student’s *t*-test.

## Figures and Tables

**Table 1:** Sequences of the primers used in RT-qPCR.

Primer	Sequence (5′-3′)
hCiCfor	GAAGTTCATCCACGACCAGAC
hCiCrev	TCGGTACCAGTTGCGCAGG
hFASNfor	GAAGGAGGGTGTGTTTGCC
hFASNrev	GGATAGAGGTGCTGAGCC
hACACAfor	GCAACCAAGTAGTGAGGATG
hACACArev	CTGTTTGGATGAGATGTGGG
hSREBP-1for	ACACCATGGGGAAGCACAC
hSREBP-1rev	CTTCACTCTCAATGCGCC
hDGAT2 for	CGAAAGCCACTTCTCATACA
hDGAT2 rev	TGCCTACTACTGCCCTCAC

## Data Availability

Not applicable.

## Supplementary Materials

The following are available online at www.mdpi.com/article/10.3390/ijms23031044/s1.
