# Supplementary material for: Quercetin Reduces Lipid Accumulation in a Cell Model of NAFLD by Inhibiting De Novo Fatty Acid Synthesis through the Acetyl-CoA Carboxylase 1/AMPK/PP2A Axis
Source: Int J Mol Sci. 2022 Jan 18;23(3):1044. doi: 10.3390/ijms23031044 (PMC8834998; doi:10.3390/ijms23031044)
Supplement: Supplementary file 1 [file ijms-23-01044-s001.zip › ijms-1511265-supplementary.pdf]

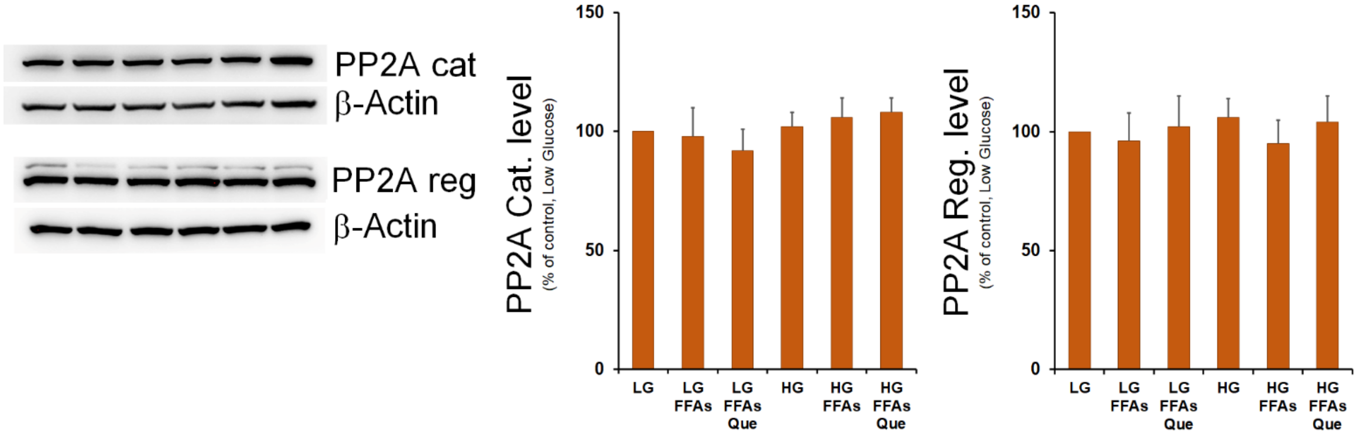

Figure S1. Expression of regulatory and catalytic subunits of PP2A in the cell model of NALFD. HepG2 cells were incubated in low glucose (LG) or in high glucose (HG), in the presence or in the absence of 0.75 mM FFAs and 5μM Que for 24 h. Total proteins were extracted from the cells and separated by SDS/PAGE. After incubation with antibodies against catalytic (PP2A Cat.) and regulatory (PP2A Reg.) subunits, the content of each protein was quantified by densitometric analysis and expressed as a percentage of control cells (LG). Values are means ± S.D. Results are representative of three different experiments.
